# Methylation kinetics and CpG-island methylator phenotyope status in colorectal cancer cell lines

**DOI:** 10.1186/1745-6150-8-14

**Published:** 2013-06-11

**Authors:** Dominik Wodarz, C Richard Boland, Ajay Goel, Natalia L Komarova

**Affiliations:** 1 Department of Ecology and Evolutionary Biology, 321 Steinhaus Hall, University of California, Irvine, CA 92697, USA; 2 Gastrointestinal Cancer Research Laboratory, Division of Gastroenterology, Baylor Research Institute, Baylor University Medical Center, Dallas, TX, USA; 3 Department of Mathematics, Rowland Hall, University of California, Irvine, CA 92697, USA

**Keywords:** Methylation kinetics, Methylator phenotype, Methylation rates, Mathematical modeling

## Abstract

**Background:**

Hypermethylation of CpG islands is thought to contribute to carcinogenesis through the inactivation of tumor suppressor genes. Tumor cells with relatively high levels of CpG island methylation are considered CpG island methylator phenotypes (CIMP). The mechanisms that are responsible for regulating the activity of *de novo* methylation are not well understood.

**Results:**

We quantify and compare *de novo* methylation kinetics in CIMP and non-CIMP colon cancer cell lines in the context of different loci, following 5-aza-2’deoxycytidine (5-AZA)-mediated de-methylation of cells. In non-CIMP cells, a relatively fast rate of re-methylation is observed that starts with a certain time delay after cessation of 5-AZA treatment. CIMP cells, on the other hand, start re-methylation without a time delay but at a significantly slower rate. A mathematical model can account for these counter-intuitive results by assuming negative feedback regulation of *de novo* methylation activity and by further assuming that this regulation is corrupted in CIMP cells. This model further suggests that when methylation levels have grown back to physiological levels, *de novo* methylation activity ceases in non-CIMP cells, while it continues at a constant low level in CIMP cells.

**Conclusions:**

We propose that the faster rate of re-methylation observed in non-CIMP compared to CIMP cells in our study could be a consequence of feedback-mediated regulation of DNA methyl transferase activity. Testing this hypothesis will involve the search for specific feedback regulatory mechanisms involved in the activation of *de novo* methylation.

**Reviewers’ report:**

This article was reviewed by Georg Luebeck, Tomasz Lipniacki, and Anna Marciniak-Czochra

## Background

Tumors are thought to emerge and progress through the activation of oncogenes and the silencing of tumor suppressor genes [1]. These processes can occur both by genetic and epigenetic processes. Mutations clearly play an important role in this context [2-4]. These range from small-scale events, such as point mutations, to larger scale events such as the loss of whole chromosomes or chromosome parts. Chromosomal loss is thought to be particularly important for the deactivation of tumor suppressor function by unmasking recessive mutations. Genetic instability [5-19] can contribute to the accumulation of mutations. Microsatellite instability speeds up the generation of small-scale mutations, while chromosomal instability speeds up the generation of larger scale mutations. Collectively, such tumor cells are referred to as “mutator phenotypes” [11]. Not only are such cells characterized by higher levels of mutations, but it has been demonstrated that such cells accumulate mutations with a faster rate or speed [10] – two measures that need not necessarily correlate with each other.

Epigenetic events are thought to be equally important, and perhaps more frequently involved in tumor initiation and progression [20-24]. Tumor cell genomes are often characterized by global hypomethylation. This has been suggested to contribute to the emergence of karyotypic instabilities as well as to the activation oncogenes. On the other hand, CpG islands are susceptible to hypermethylation, and when it occurs in the promoter, it is associated with gene silencing and can promote the deactivation of tumor suppressor genes. Similar to the mutator phenotype concept, a “methylator phenotype” concept has emerged to account for the tendency to observe patterns of hypermethylation in certain cell lines. Tumor cell lines that are characterized by relatively high levels of CpG island methylation have been called CpG island methylator phenotypes (CIMP cells), which sets them apart from non-CIMP cells that are characterized by lower levels of CpG island methylation [25-28].

The term “methylator phenotype” implies that relevant loci in such cells become methylated faster. A recent study [29,30] demonstrated reduced fidelity in replicating methylation patterns of CpG islands in CIMP gastric cell lines compared to non-CIMP lines, mostly caused by *de novo* methylation. The methylated status of CpG sites was more stably maintained than the unmethylated state. This could lead to the methylation of entire CpG islands in experiments that allowed clonal expansion of cells. These experiments concentrated on the methylation kinetics in situations when the genome was already highly methylated.

In order to obtain a more detailed understanding of the differences between CIMP and non-CIMP cells, we aimed to investigate the methylation kinetics in cells that were partially de-methylated. This was achieved by measuring the *de novo* methylation rate following 5-aza-2’deoxycytidine (5-AZA) treatment in 2 CIMP and 2 non-CIMP cell lines, using a combination of experimental and mathematical approaches. The analysis was performed in the context of 2 loci (ALU and LINE-1), and the 5 genes APC1, RASSF2-1, HPP1, SFRP2, and MGMT. We made the surprising finding that CIMP cells lines were characterized by relatively slow rates of *de novo* methylation, while non-CIMP cell lines were characterized by relatively fast rates of *de novo* methylation. Further, while CIMP cells lines accumulated methylation slower, they started the re-methylation process relatively early and accumulated methylation steadily following 5-AZA-treatment. On the other hand, non-CIMP cell lines typically showed a time delay after 5-AZA treatment before displaying a burst of relatively fast methylation kinetics. We hypothesize that these kinetics can be explained by feedback-mediated regulation of *de novo* methylation activity, which is corrupted in CIMP cells. A mathematical model shows that these assumptions can give rise to our experimentally observed methylation kinetics.

## Results

### Confirmation of CIMP status

Based on observed patterns of hypermethylation, cell lines SW48 and RKO have been designated as CIMP cell lines in the literature [31]. The cell lines HT29 and HCT116 are thought to be non-CIMP cell lines [31]. We aimed to confirm these classifications in the context of our experiments. Hence, the baseline methylation level of the four cell lines was measured with respect to the 7 different sites: ALU, Line-1, APC1, RASSF2-1, HPP1, SFRP2, and MGMT. Results of these measurements are consistent with the previously established CIMP classification. The baseline methylation levels of HCT116 and HT29 are generally lower than those for SW48 and RKO.

### Observed patterns of *de novo* methylation

**Figure 1:**
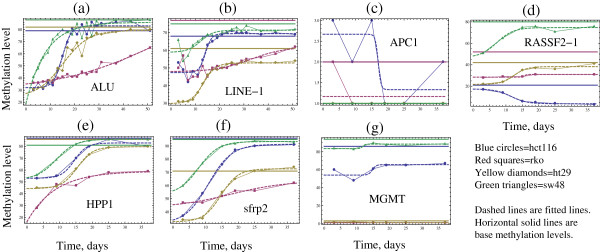
**Methylation time-series for four cell lines, for 7 sites.** The results of experimental measurements are presented by connected points, and the fitted functions are given by dashed lines. The cell lines are color-coded, and the base methylation level for each cell line is presented by a horizontal line of the appropriate color. The pannels (**a**-**g**) correspond to the 7 different sites.

The cells were treated with 5-AZA for the purpose of demethylation. After treatment, we examined the temporal process of re-methylation with respect to the 7 sites. At several time-points post-treatment, we measured the percentage of methylation for each site for each cell line by quantitative pyrosequencing assays. The results of these measurements are presented in Figure 1. There, each of the 7 plots (a-g) corresponds to a different site, which is marked on the corresponding graph. The four lines on each graph correspond to the four cell lines, which are color-coded. The measured methylation level (as percentage) is plotted against time post-treatment. The baseline methylation levels for the cell lines are presented by horizontal lines of the corresponding color.

We can see that qualitatively, the majority of remethylation curves start at a level lower than their base methylation level, and then climb up with visibly different slopes. Some of the curves reach saturation (which interestingly may or may not coincide with the measured baseline methylation level), while others continue to climb during the whole duration of the experiment. Another noticeable feature of some of the curves is the presence of a time-delay. In fact, the majority of the experimental runs can be assigned to one of two different groups, as shown in Figure 1. In one group, the methylation level starts climbing up immediately upon the cessation of 5-AZA treatment. An example of such behavior is exhibited by line SW48 (the green line in Figure 1(a)). In the other group, there is a certain time-delay between the cessation of 5-AZA treatment and the point where the methylation process picks up. For example, the blue line (HCT116) in Figure 1(a) climbs up between time-points approximately 10 and 25. For the first 10 days post 5-AZA treatment, the process of remethylation is slow to gain momentum.

### Quantifying the *de novo* methylation kinetics

In order to quantify these data and to extract information about methylation rates, we performed fitting of these data with the function *f*(*t*) = *y*_0_ + *y*_1_ tanh(*mt* − *b*). This function is characterized by 4 parameters. Parameter *m* (days^-1^) measures the methylation rate; the dimensionless parameter *b* characterizes the time-delay until the *de novo* methylation process starts to gain momentum, as described above; parameters *y*_*0*_ and *y*_*1*_ are related to the initial and target methylatin level, with *y*_0_ − *y*_1_ tanh(*b*) being the initial methylation level (after the 5-AZA treatment), and *y*_0_ + *y*_1_ the target methylation level. The choice of this functional form was dictated by the patterns seen in the methylation time-series and described above. Function *f(t)* is one of many possible functions capable of accounting for the two types of methylation patterns, with and without delay. The exact mathematical form of this function is unimportant, as long as the following requirements are met: (a) the function has the ability to capture a climb (gradual or step-like) from one level to another level and (b) has parameters which can be extracted to characterize the slope of the climb (the *de novo* methylation rate) and the amount of time delay. In the function *f(t)* chosen here, parameter *m* measures the *de novo* methylation rate, and parameter *b* characterizes the time-delay.

Figure 1 presents the results of fitting the function *f(t)* to the data, which for each case is given by a dashed line of the appropriate color. Out of the total of 4x7 =28 experimental runs, we were able to successfully fit 18: these include all the cell-lines in the context of ALU, LINE-1, HPP1, and SFRP2, as well as lines HT29 and SW48 in the context of RASSF2-1. We will refer to these experimental runs as “successful runs”. For gene APC1, the base methylation levels were too low to be included in the anlaysis. For gene RASSF2 − 1, cell lines HCT116 and RKO failed to re-methylate upon 5-AZA treatment (the measured methylation levels stayed more or less constant for RKO, and they actually showed a decline for HCT116). For gene MGMT, lines RKO and HT29 did not have a significant base methylation level, and lines SW48 and HCT116 did not exhibit a significant climb which would allow us to assess the methylation rate with confidence (in the case of SW48, the 5-AZA treatment did not lead to a significant decrease of the methylation level compared to the base level, and in the case of HCT116, the pattern of methylation was erratic leading to the failure of the fitting procedure). Therefore, we will proceed with the analysis of the data from the 18 successful runs.

**Figure 2:**
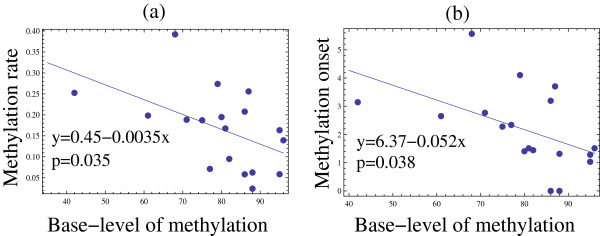
**Scatter plots of some methylation parameters extracted from the 18 successful experimental runs, and their correlations**. (**a**) A scatter plot of the methylation rate vs the base methylation level. (**b**) A scatter plot of the (dimensionless) methylation onset parameter, *b*, vs the base methylation level. The linear model together with the p-value are marked on the plots.

**Figure 3:**
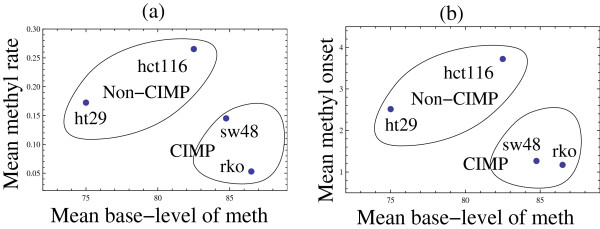
**Scatter plots of the mean characteristics presented in Figure **2**for each cell line across the loci (a).** The mean methylation rate vs the mean base methylation level. (**b**) The mean (dimensionless) methylation onset parameter vs the mean base methylation level. Each point corresponds to one cell-line, marked in the plots. Also, the CIMP status of each cell line is indicated.

Fitting the function *f(t)* to the data yielded the numerical values of the parameters *m* (remethyation rate) and *b* (the dimensionless methylation onset). We will first focus on the remethylation rate. The parameter *m* characterizes the “steepness” of the slope of the methylation time-series in the regions where *de novo* methylation process takes place (and it does not capture other aspects of the methylation process). Figure 2(a) presents a scatter plot of the measured re-methylation rates versus the baseline methylation level for the 18 experients. We performed a linear regression analysis of this scatter plot and determined that the methylation rate negatively correlates with the base methylation level, with the Pearson rank correlation of −0.499 and the Spearman rank correlation of −0.574 (for the sample size of n = 18). This correlation is significant, with the *p*-value of 0.035 (or *p* = 0.013 if using the Student’s t-distribution with the Spearman rank correlation). This surprising result suggests that the rate at which the methylation level climbs up after 5-AZA treatment is the lowest for the cell lines with the highest base-level of methylation. This can be clearly seen in Figure 3(a), where we plot the mean average methylation rate for the four cell lines against their mean base methylation level. In the figure, we group the four cell-lines investigated into two groups: lines HT29 and HCT116 comprise the non-CIMP group, while lines SW48 and RKO are classified as CIMP. This grouping is consistent with the characterization these cell lines received in the literature [31], and also corresponds to the higher base methylation levels for the CIMP cells. We can see that lines HT29 and HCT116 (which exhibit lower base methylation levels compared to their CIMP counterparts) are characterized by higher *de novo* methylation rates. Thus, CIMP cell lines show slower rate of re-methylation, while non-CIMP cell lines show faster rates of re-methylation.

To examine this phenomenon more closely, we performed further data analysis. While parameter *m* only provides information on how steeply *de novo* methylation curves climb up, it does not reflect the presence or the absence of a time-delay in the onset of the re-methylation process. To quantify these differences in a systematic way, we look at parameter *b*, which yields the dimensionless onset for remethylation. It turns out that there is a significant negative correlation between the base methylation level, and the onset parameter, *b* (the *p*-value is 0.038), see Figure 2(b) and 3(b). There is also a very strong (with *p = 2x10*^*-6*^) positive correlation between the re-methylation rate, *m*, and the onset parameter, *b* (not shown). This suggests that the *de novo* methylation rate and the onset parameter (as specified by the function *f*) vary together, and a better description of the observed phenomena is provided by a function $\tilde{f}\left(t\right)=y_{0}+y_{1}\tanh\left(m\left(t-b'\right)\right)$, where parameter *b’ = b/m* measures the delay time in days. The time-delay *b’* does not show a significant correlation with the base methylation levels (not shown). The function $\tilde{f}\left(t\right)$ assumes explicitly that re-methylation time-series that climb faster, tend to experience a longer delay between the 5-AZA treatment cessation and the onset of *de novo* methylation.

To summarize this analysis, we can say that the CIMP phenotypes, which are characterized by higher base methylation levels, tend to show a slower *de novo* methylation rate, but experience a steady climb in methylation levels which starts soon after the cessation of 5-AZA treatment. In contrast, non-CIMP cell lines with a lower base methylation level tend to exhibit a certain delay in re-gaining their methylation status, followed by a relatively rapid increase in methylation levels. Roughly speaking, non-CIMP phenotypes have a spurt a of relatively rapid methylation increase following a relatively long delay. The methylator phenotypes start increasing their methylation levels relatively quickly and steadily, albeit slowly.

To gain further insights, we also performed a systematic correlation analyses of several other characteristics (see Appendix), which did not reveal any further statistically significant correlations. We also tested the hypothesis that *de novo* methylation rates are correlated with cellular kinetic parameters of the dividing cell lines, such as their division and death rates. No significant patterns have been found.

### A mathematical model to explain observations

A key finding from our quantitative analysis was that CIMP cells tended to start the methylation process immediately upon cessation of 5-AZA treatment, but did so relatively slowly, while non-CIMP cells showed a relatively fast burst of re-methylation but only after a certain time delay following treatment cessation. The existence of a time delay before a phase of accelerated re-methylation could indicate the existence of a negative feedback loop in the regulation of the *de novo* methylation process. The basic idea is as follows. If methylation levels in the genome are around a certain homeostatic setpoint, *de novo* methylation ceases to occur due to negative feedback, and only maintenance methylation takes place. On the other hand, if the methylation levels are significantly reduced, e.g. following 5-AZA treatment, release of negative feedback induces appropriate DNA methyltransferase (MTase) activity. The process of activation typically is not instantaneous but requires the interactions among several factors, leading to a delay [32-34]. MTase activation leads to a burst of *de novo* methylation, which is shut down again through negative feedback as methylation levels in the genome recover. On the other hand, it can be hypothesized that in CIMP cells this feedback regulatory mechanism is corrupted and the appropriate MTases are constantly active at a relatively low level, leading to slow but continuous *de novo* methylation, as reported experimentally [29,30]. According to this scenario, re-methylation of CIMP cells would commence without a delay following 5-AZA treatment, and would proceed with a relatively slow rate because of the continuous activity of MTase. The exact MTase responsible for CpG island *de novo* methylation in cancer cells is debated. In the context of non-cancerous cells, it is thought that DNMT1 contributes mainly to maintenance methylation, while *de novo* methylation activity is ascribed to DNMT3a and DNMT3b [35,36]. Work in human cancer cell lines, however, demonstrated that DNMT1 can exhibit *de novo* methylation activity for CpG islands [37], and DNMT1 has been shown to be up-regulated in different tumor types [38-40].

To investigate whether this hypothesis is consistent with the observed experimental patterns, we construct a mathematical model of *de novo* methylation in the context of negative feedback regulation of MTases that are responsible for *de novo* methylation. The model takes into account the following variables. The methylation level of the loci in question is denoted by *x*. The methylation level of loci that drive the negative feedback is denoted by *w*. These remain hypothetical loci for now. The molecular processes that regulate *de novo* methylation are not well understood [33,41], although feedback mechanisms have been implicated in the regulation of DNMT1 [42]. It has been suggested that the methylation status of regulatory elements of DNMT1 determines the activity of this MTase, which could result in negative feedback. This element has been shown to be highly methylated in somatic tissues, and unmethylated in a mouse adrenal carcinoma cell line [42], consistent with the notions explored here. For simplicity, we will refer to such regulatory elements as “feedback sensors”, without assuming a particular mechanism that underlies feedback. Our model generally assumes a “sensor” that reduces and shuts down MTase activity if methylation levels in the genome rise towards some homeostatic level. The model can be adjusted as specific biological information becomes available. MTase activity that is required for *de novo* methylation is denoted by *y*_*n*_. It is assumed that activation of MTase requires the interactions of different signaling components, which are denoted by *y*_*i*_, where *i = 1…n-1*. The model is given by the following set of ordinary differential equations, which describe the time-evolution of these variables.

$$\begin{array}{l} \frac{\mathrm{dx}}{\mathrm{dt}}=\lambda y_{n}\left(1-\frac{x}{k_{1}}\right)-a_{1}x,\\ \frac{\mathrm{dw}}{\mathrm{dt}}=\gamma y_{n}\left(1-\frac{w}{k_{2}}\right)-a_{2}w,\\ \frac{dy_{1}}{\mathrm{dt}}=\eta_{\mathrm{prod}}-qy_{1},\\ \frac{dy_{i}}{\mathrm{dt}}=qy_{i-1}-qy_{i},1<i<n,\\ \frac{dy_{n}}{\mathrm{dt}}=qy_{n-1}-by_{n}. \end{array}$$

The locus in question is methylated in the presence of active MTase (*y*_*n*_) with a rate *λ*. It is assumed that *k*_*1*_ methyl groups can be added. During 5-AZA treatment, de-methylation occurs with a rate *a*_*1*_. Similarly, *de novo* methylation of feedback sensors occurs with a rate *γ* in the presence of MTase, *y*_*n*_, and 5-AZA treatment causes de-methylation of these loci with a rate *a*_*2*_. The maximum methylation level of feedback sensors is given by *k*_*2*_. Activation of MTase, *y*_*n*_, occurs via a signaling cascade, *y*_*i*_. Regulation occurs in the first element of this signaling cascade, *y*_*1*_, which is produced with a rate *η*_*prod*_. Details of this production term depend on the nature of the cell line. For non-CIMP cells, we assume the presence of negative feedback. Thus, if the methylation levels of feedback sensors lie below a threshold, *c*, production occurs with a rate *η(c-w)*. If the methylation level of feedback sensors rises above this threshold, the rate of production is set to zero. On the other hand, for CIMP cells, it is assumed that production of *y*_*1*_ occurs at a constant rate *η*. Once *y*_*1*_ is produced, it induces MTase activity through interactions with elements of the signaling cascade, *y*_*i*_. Finally, MTase activity decays with a rate *b*.

We will concentrate on the parameter regime where the MTase activity is relatively short-lived in the absence of the activation signals in the model. That is, the parameter *b* is sufficiently large. This ensures that when the activation signal is switched off, *de novo* methylation ceases without significant delay. Model properties will be described first for non-CIMP cells and then for CIMP cells under this assumption.

**Figure 4:**
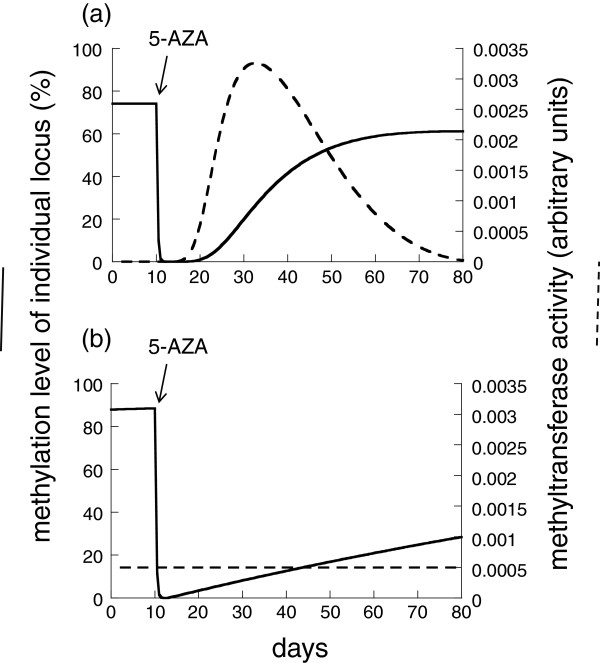
**Computer simulation of the re-methylation kinetics in (a) non-CIMP and (b) CIMP cells, according to the mathematical model described in the text.** 5-AZA treatment is indicated by the arrow. The solid line represents the methylation level of an individual locus, *x*, while the dashed line represents *de novo* MTase activity, *y*_*n*_. Parameters were chosen as follows. (**a**) *λ = 0.4; k*_*1*_ *= 0.04; a*_*1*_ *= 4; g = 80; k*_*2*_ *= 100; a*_*2*_ *= 0.4 η = 2; c = 10; q = 0.8; b = 4000*; (**b**) same, but *η = 2*.

For non-CIMP cells, the methylation of feedback sensors, *w*, always rises towards a level given by the parameter *c*, after which the negative feedback kicks in and *de novo* methylation stops. Hence, the methylation of *w* remains constant at this level. On the other hand, the degree to which individual loci become methylated before *de novo* methylation is shut down by negative feedback depends on initial conditions, as explained below. Figure 4a has been generated by starting the computer simulation with a completely un-methylated cell and allowing the genome to become methylated. In this case, the individual methylation of locus *x* stabilizes around 75%. This stable state is shown in Figure 4a before the start of 5-AZA treatment. Then, 5-AZA treatment is initiated in the simulation and maintained for 72 hours, after which treatment stops. Following the simulated 5-AZA treatment, both the site of interest, and the feedback sensors, become demethylated. However, the feedback sensors retain a higher level of methylation. This is compatible with the notion that different sites in the genome de-methylate at different rates in response to 5-AZA treatment. After cessation of 5-AZA treatment, after a certain time delay, active MTase levels rise and *de novo* methylation increases at a relatively fast rate, as seen in the experimental data. Over time, the methylation level stabilizes. In the simulation of Figure 4a, it stabilizes at a lower level compared to the base methylation level, as observed in some of the experimental patterns described here. This is a consequence of the assumption that the methylation level of feedback sensors, *w*, was reduced to a lesser degree than the methylation level of the locus under consideration, *x*. Therefore, upon re-methylation, feedback sensors reach their homeostatic set-point and shut down MTase activity before methylation of the locus under consideration has reached its pre-treatment base level. In general, the degree to which given loci become re-methylated following 5-AZA treatment depends on the exact state of the cell after treatment is complete. It can become less methylated, or it can achieve the same amount of methylation seen before treatment. Restoration of pre-treatment methylation levels is likely either if the amount of methylation of feedback sensors is reduced more during 5-AZA treatment, or of the loci in question become de-methylated to a lesser extend during treatment.

For CIMP cells, different dynamics are observed (Figure 4b). Before 5-AZA treatment, the methylation level of the locus *x* is not stable but rises at a slow rate. This is the consequence of the assumption that MTase activity is constantly on at relatively low levels and that feedback regulation is corrupted. As before, the simulation assumes 5-AZA treatment for 72 hours. Re-methylation commences instantly and occurs at a relatively slow rate, as observed in the experimental data. Again, the reason is that MTase activity is constantly on. Thus, after de-methylation, it does not have to be activated and hence re-methylation starts immediately. Similarly, because feedback regulation is corrupted, low levels of methylation do not induce a sharp rise in the methylation rate.

In summary, the mathematical model reproduces the key phenomena found in the data: Following 5-AZA treatment, non-CIMP cells re-methylate with a faster rate following a certain time delay, while CIMP cells start re-methylation immediately, although at a slower rate. Moreover, in agreement with the data, the model predicts that in non-CIMP cells, individual loci can re-methylate to levels that are lower than those found before 5-AZA treatment.

## Discussion

In this paper, the *de novo* methylation kinetics of different loci were investigated in CIMP and non-CIMP colon cancer cell lines following 5-AZA induced de-methylation of these cells. The analysis showed that while CIMP cells start re-methylating immediately after cessation of 5-AZA treatment, the rate of re-methylation is relatively slow. On the other hand, non-CIMP cells tended to show a delay before re-methylation commenced after 5-AZA treatment. However, once the process started, re-methylation occurred significantly faster than in the CIMP cells. Interestingly, the methylation levels of the investigated loci did not always return to pre-5-AZA levels, but converged to a new, lower level in several cases.

The slower re-methylation kinetics observed in CIMP cells comes as a surprise given the observed hyper-methylation of CpG islands in these cells. It also appears to be at odds with a recent study which investigated patterns of *de novo* methylation in CIMP and non-CIMP gastric cancer cell lines [29,30]. In this study, however, cells were not de-methylated before measuring the kinetics. CIMP cells were characterized by a higher *de novo* methylation rate than non-CIMP cells. This could lead to the methylation of entire CPG islands during clonal expansion.

To explain our observed kinetics and to reconcile them with the observed increased *de novo* methylation rates found in CIMP gastric cancer cell lines [29,30], we invoked the hypothesis of feedback-regulated activity of *de novo* methylation. If methylation levels are relatively low, maximal MTase activity is attained in order to re-methylate the cell; *de novo* methylation stops if the degree of methylation of feedback sensors reaches a defined level. In addition, we hypothesize that this negative feedback is corrupted in CIMP cells. In this case, low level of CIMP activity occurs constantly. When these assumptions are formulated into a mathematical model, the key findings reported here can be reproduced, including the differences in re-methylation kinetics and the observation that maximal methylation levels can be lower post 5-AZA treatment than before treatment. Further, this hypothesis predicts fundamentally different observations in cells that have been de-methylated and in cells characterized by physiological levels of CpG island methylation. In de-methylated cells, the release of negative feedback gives rise to the result that methylation rates are significantly faster in non-CIMP compared to CIMP cells. With physiological levels of methylation, *de novo* methylation rates are faster in CIMP cells than in non-CIMP cells, in which feedback has largely shut down the process of *de novo* methylation.

The mechanisms that are responsible for regulating the activity of *de novo* methylation are not well understood. Data suggest a dynamic interplay between different posttranscriptional modifications [33], and the occurrence of negative feedback has been suggested in the context of DNMT1 [42]. Since specifics are currently lacking, our model tried to capture this complexity by the presence of a multi-component signaling cascade that participates in the induction of *de novo* methylation activity when methylation levels are reduced.

The proposed model is not the only possible explanation of the observed behavior. Different combinations of feedback loops, e.g. such as proposed in [43] could be consistent with the behavior exhibited by the cell lines. The purpose of the present model is to demonstrate that feedback loops can be accountable for the observed counter-intuitive behavior. Further investigation of the mechanisms of methylation kinetics will inform the exact topology of the feedbacks. Other approaches such as stochastic Markov models (e.g. [44-46]) can be very useful, especially given a very high degree of heterogeneity in the cell lines’ behavior. As more specific information becomes available regarding regulatory processes, the models can be updated and modified to provide a less phenomenological description of these intra-cellular dynamics.

Another factor that needs to be taken into consideration is the possibility that the 5-AZA treatment significantly altered the properties of the cells, and that this could have contributed to the slower re-methylation rates observed in CIMP cells. In fact, we found the trend that lower re-methylation rates were observed when the degree of 5-AZA-induced de-methylation was stronger. The correlation, however, was not statistically significant. The overall effect of de-methylation on the gene expression profile of cells is likely to be highly complex and needs further investigation. There is an indication that DNA methylation status alone cannot account for gene expression patterns, but that a coordination of DNA methylation and histone modifications can determine transcriptional status [47].

## Conclusions

In conclusion, the faster rate of re-methylation observed in non-CIMP compared to CIMP cells in our study could be a consequence of feedback-mediated regulation of MTase activity. Thus, in non-CIMP cells, release of feedback causes a burst of *de novo* methylation activity. In CIMP cells, this burst does not occur. Instead, the re-methylation kinetics are governed by the constant low level activity of MTase. According to this hypothesis, the situation is reversed once the genome has accumulated a certain amount of methylation. In this case, *de novo* methylation ceases to occur in non-CIMP cells, while it persists at some level in CIMP cells. Testing this hypothesis will involve the search for specific feedback regulatory mechanisms involved in the activation of *de novo* methylation. A better understanding of the differences between CIMP and non-CIMP cancer cells is relevant both from a basic scientific point of view, and also from a treatment perspective. Our data and previously published work [29,30] indicate that CIMP and non-CIMP cells do not show a straightforward and easy to interpret difference in methylation rates, i.e. CIMP cells are not simply characterized by a faster rate of methylation, analogous to the faster mutation rate seen in mutator phenotypes. The relationship between CIMP status and the rate of methylation is complex, seems to depend on the exact methylation status of the cell, and is likely driven by complex regulatory mechanisms. This in turn has implications for understanding the definition of the CIMP status. Understanding those mechanisms will be important to assess the consequences of treatment concepts which aim to reduce hypermethylated states in cancer cells, thus possibly reversing some of the malignant phenotypes displayed by those cells [48].

## Methods

### Cell culture

Four human colorectal cancer (CRC) cell lines including HCT116 (microsatellite unstable or MSI), HT29 (microsatellite stable or MSS), RKO and SW48 (CpG Island methylation phenotype or CIMP) were obtained from the American Type Culture Collection (ATCC, Manassas, VA). Cells were cultured in IMDM medium (Invitrogen, Rockville MD) under standard conditions with 10% fetal bovine serum with 5% CO_2_ at 37°C and the negative status for mycoplasma infection was repeatedly confirmed.

### 5-aza-2’deoxycytidine (5-AZA) treatment

All cell lines were treated with 5-AZA in order to allow global CpG demethylation in four different cancer cell lines. Briefly, 5-AZA was dissolved in PBS (pH 7.5) to 5 mM concentration and small aliquots were kept frozen at −20°C. Twenty four hours after seeding equal number of cells in 10 cm petri dishes, all four cell lines were treated with 2.5 μM 5-AZA (Sigma-Aldrich, MO, US) for 72 h. After the completion of 72 hour treatment, each dish was replaced with fresh culture medium without 5-AZA and the cells were cultured and harvested at specified time points. The cells were subsequently trypsinized, carefully counted and subsequently subjected to DNA extraction for methylation analysis.

### DNA Extraction, Bisulfite Modification and methylation analysis

DNA extraction was performed with the DNeasy Blood & Tissue kit (Qiagen, Valencia, CA) according to manufacturer’s instructions. DNA was modified with sodium-bisulfite using the EZ Methylation Gold Kit (Zymo Research, Orange, CA) as previously described [49]. Thereafter, the methylation status of various tumor suppressor genes including MGMT, APC, SFRP2, RASSF2 and HPP1 was analyzed using quantitative bisulfite pyrosequencing in a PCR reaction containing bisulfite modified DNA, HotStarTaq polymerase, forward primer, biotinylated reverse primers and water. In addition, the methylation status of Long interspersed nuclear element-1 (LINE-1) methylation was used as a surrogate marker for genome-wide methylation. Previous studies have shown that LINE-1 methylation correlates with global DNA methylation [50,51]. Following PCR amplification, four microliters of PCR product were added to 38 μl of binding buffer (Biotage, Uppsala, Sweden), 2 μl streptavidin sepharose high-performance beads (GE Healthcare, Buckinghamshire, England) and 36 μl of sterile water. Single-stranded biotinylated templates were isolated using the PyroMark Vacuum Prep WorkStation (Biotage). The products were dispensed into 96 well plates containing 0.36 μl 10 μM sequencing primer and 11.64 μl annealing buffer (Biotage) at 80°C for 3 min, and then placed at room temperature for 10 min. Pyrosequencing reactions were carried out in the Pyro-Mark MD (Qiagen, Hilden, Germany) using PyroGold reagents and results were analyzed using pyro Q-CpG Software (Biotage).

### Systematic correlation analysis

**Figure 5:**
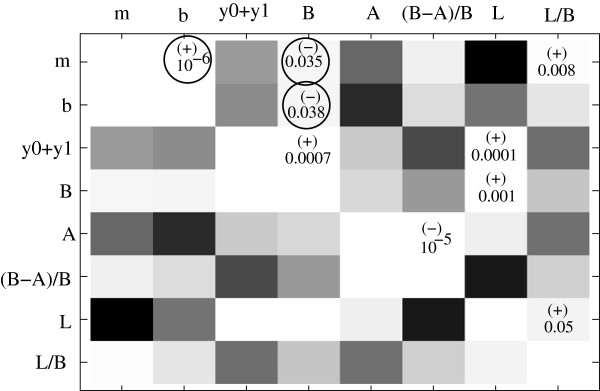
**Results of the correlation analysis for pairs of parameters.** The lighter color corresponds to lower p-value of the corresponding correlation. The *p*-values for the strongest correlations are indicated, together with a (+) for positive correlations or (−) for negative correlations.

To search for correlations in the observed remethylation patterns, we split measurable parameters into two groups. The first group comes from the fitting procedure of the function *f(t)*, and the corresponding parameters are related to the four values *m* (the rate of *de novo* methylation), *b* (the delay), *y*_*0*_ , and *y*_*1*_ (connected to the initial and final methylation level where the fitted function levels off). The second group contains those values that can be read directly from our measurements, without fitting the model. These are the base methylation levels (denoted here by *B*); the methylation level to which the cell lines drop upon the 5-AZA treatment (denoted by *A*); the percentage of methylation reduction as a result of 5-AZA treatment, *(B-A)/B*; the level of methylation on the last day of the experiment (*L*) as well as its relative value (*L/B*), etc. We performed linear regression analyses for all pairs of these parameters within and between the two groups. Figure 5 presents the results of this analysis. There, we show a table of *p*-values revealed by the correlation analysis for all pairs of parameters. Lighter squares correspond to smaller values of *p*. For example, all the diagonal elements have *p = 0*. The p-values that correspond to statistically significant correlations are marked on the upper right half of the diagram (the lower left half is a mirror image of the upper right half). Whether the correlation is positive or negative is marked by a plus or a minus sign above the *p*-value. This systematic analysis revealed the three correlations described above, which are denoted by circles in Figure 5. Some other correlations can be observed, which are expected. These include: (1) the positive correlation between *m* and *L/B*, which is a combination of two effects: (i) the negative correlation between *m* and *B*, and (ii) the fact that functions with a faster growth rate will recover more of their base methylation level by the last day of the experiment; (2) a positive correlation between the predicted (*y*_*1*_ *+ y*_*0*_) and the observed (*L*) final level of the re-methylation process; (3 and 4) the positive correlation between the base level (*B*) and the final level of methylation, both predicted (*y*_*1*_ *+ y*_*0*_) and observed (*L*); (5) the negative correlation between *A* and *(B-A)/B*; (6) the positive correlation between *L* and *L/B* (the last two correlations simply follow from the definitions of the relative values, *(B-A)/B* and *L/B*). No other statistically significant correlations are found.

Finally, we tested the hypothesis that *de novo* methylation rates are correlated with cellular kinetic parameters of the dividing cell lines. This was accomplished in the following way. For the four cell lines under investigation, we performed cell counts of both live and dead cells at several time-points. The experiment was then repeated with cells treated with 5-AZA. We subsequently extracted the information on the rates of cell divisions and deaths from the data. To do this, we first fitted the function *x*(*t*) = *Ae*^*gt*^ to the time-series of the live cell counts, to determine the net growth rate, *g*, by means of the standard least square procedure. The net growth rate is comprised of the division rate, *b*, and the death rate, *d*. To tease out these two rates, we used the dead cell counts. Namely, we assumed that between times *t*_*i-1*_ and *t*_*i*_, the live (*x*) and dead (*y*) cells satisfy the following equations,

$$\begin{array}{l} \dot{x}=\mathrm{gx},x\left(t_{i-1}\right)=Ae^{gt_{i-1}},\\ \dot{y}=\mathrm{dx},y\left(t_{i-1}\right)=0. \end{array}$$

**Figure 6:**
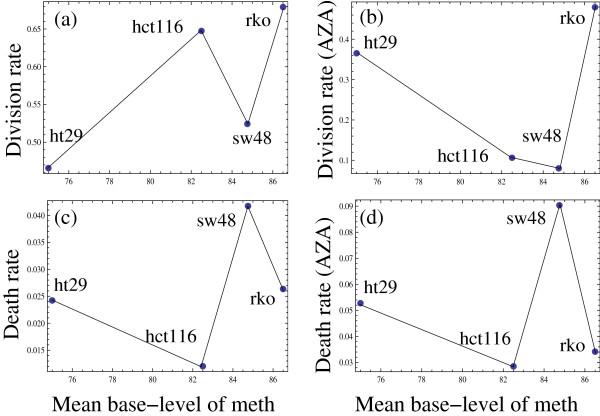
**Scatter plots of the measured cells’ growth kinetic parameters vs their base methylation levels**. (**a**) The division rate in the absence of 5-AZA, (**b**) the same for 5-AZA-treated cells, (**c**) the death rate in the absence of 5-AZA, (**d**) the same for 5-AZA-treated cells. Each dot corresponds to a particular cell-line, which is marked on the plot.

The initial condition for the number of dead cells corresponds to the fact that the dead cells are removed after counting at each time-step. The parameters *A* and *g* are known from the previous fitting procedure. The solution for *y* is then given by $y\left(t_{i}\right)=\frac{\mathrm{dA}}{g}\left(e^{gt_{i}}-e^{gt_{i-1}}\right)$*,* and the unknown value of *d* can be found from the time-series of the dead cell counts by the usual least squared procedure. Finally, the division rate is given by *b = g + d*. Results of these calculations are presented in Figure 6, which shows the division and death rate of the four cell lines as a function of their base methylation level. Results are presented both for control cells and for 5-AZA-treated cells. No discernable pattern can be seen.

## Reviewers’ reports

### Reviewer 1: Georg Luebeck and Bill Hazelton

This manuscript presents interesting data and a model for the time course of methylation of five genes and two retrotransposons in each of four colorectal tumor cell lines following demethylation by 5-aza-2’deoxycytidine (5-AZA). Two of the four tumor cell lines were classified as CpG island methylator phenotypes (CIMP)s, and the other two cell lines as non-CIMP, which typically have lower levels of CpG island methylation. The data come from measuring the initial level (percentage) of methylation in 28 experiments (7 genes X 4 cell lines), then demethylation using 5-AZA, following by repeated measurements of the time course of methylation over 40–50 days. Significantly, but contrary to initial expectations, methylation generally increased sooner but at a slower rate among CIMP cells compared with non-CIMP cells.

Somatic maintenance of DNA methylation in dividing cells has been shown to be dependent on a system of Dnmt methyltransferases, which are processive, interactive, and are linked to DNA replication. Stochastic Markov models of this system have been developed previously and can be found in the literature (e.g., see Otto and Walbot, 1990; Genereux et al., 2005). Along these lines, Sontag et al. (2006) developed an interactive Markov state model which embraces the full set of states (un-methylated, hemi-methylated upper (lower) strand, fully methylated) to study the interplay between maintenance (Dnmt1) and de novo methyltransferases (Dnmt3a/b) in maintaining stably both un-methylated and methylated CpG-rich regions. There are two intriguing findings from these models: (1) the fidelity of maintenance methylation is poor (~95%) and without the presence of a significant contribution of random de novo methylation leads to de-methylation. (2) DNA methylation patterns appear to be bi-modal, i.e., CpG islands in individual genomes are either grossly unmethylated or more or less fully methylated at the genome level. An exploration of the effect of 5-AZA on these dynamics (post-replicative CpG methylation via Dnmt’s and natural de-methylation during DNA replication) would be very interesting but may require advanced sequencing technology to resolve regions-specific methylation states in individual cells, providing information that cannot be obtained from studying methylation across cells in the form of averages. Because 5-AZA acts via suppression of Dnmt1 (likely through enhanced degradation), the first generation daughters that end up hemi-methylated after 5-AZA treatment are likely to remain in the cell pool. It is not clear how selection figures into this. For example, does recovery after treatment select the hemimethylated daughter cells, which arguably seem more intact than unmethylated progeny?

***Authors’ response***: *Thank you for this comment, we have discussed these references in the text of the revised manuscript.*

In the original version of the paper, methylation levels were modeled using a phenomenological approach. Specifically, a hyperbolic tangent function, f(t) = y0 + y1 tanh(mt-b), was used to describe the methylation level following 5-AZA treatment at time t. m is the methylation rate, y0 + y1 is the target (final) methylation rate, y0 - y1 is described as the initial methylation level (after 5-AZA treatment), and b is described as the time delay (in days, as stated at the bottom of p 9) until the de novo methylation process starts to gain momentum. This description and/or function requires some clarification which the authors provide in their revision. Briefly, setting t = 0 in the original equation, the initial methylation level at the time of 5-AZA treatment is f(0) = y0 + y1 tanh(−b), which is not equal to the stated initial level, y0 – y1 , unless the ‘time delay’ b approaches infinity.

***Authors’ response***: *Thank you, we corrected this in the text.*

Second, the product mt in the argument of tanh(mt-b) is dimensionless (as required) since it is the product of a rate with time, and likewise quantity b must also be dimensionless. However, b is repeatedly called the time delay, and on p 9 is stated to be “the time until methylation onset (in days)”. This problem with units could be corrected by writing the function as f(t) = y0 + y1 tanh(m(t-b’)), where now b’ does have dimensions of time, the argument of tanh is properly dimensionless, and the actual time delay is b’ = b/m showing that b in the formula as written is a scaled time. This may appear to be a trivial point, but not recognizing b as a scaled time appears to have led to two problems, the first being that the plot of b (on the y-axis of Figure 2b, labeled “Methylation onset”) ranges between about 1–6, and does not match the time delays plotted for the actual data in Figure 1, where the time delay (time to maximum slope of the hyperbolic tangent) ranges from about 1–30 days. These differences are reconciled through scaling by dividing b by the factor m, which is typically in the range of 0.01 – 0.40. The second place this causes problems is that it introduces a strong correlation between methylation rate m and “onset time” b. This very strong correlation is noted at the top of p 10, without explanation. Use of the actual delay time b’ would appear to markedly decrease this correlation, although it may not remove it completely.

***Authors’ response***: *This is a very important observation, which was incorporated in the new version of the manuscript. The parameter b’ (measured in days) turned out to be un-correlated with the base methylation rate. Moreover, the very strong (p = 10*^*-6*^*) correlation between the dimensionless onset parameter b and the methylation rate m suggests that they vary together, and the process is best described by the function the referees suggested. In the new version of the paper, we actually start with the old formulation of the fitting function, and arrive at the corrected formulation following the correlation analysis. The confusion regarding the dimensionality of the parameter b has been cleared.*

There are several interesting features evident in these data. Several such features, as noted in the manuscript, include non-CIMP cells undergoing a relatively fast rate of methylation following a time delay, while CIMP cells methylate slower with smaller or no delay; and that the rate at which methylation climbs up after 5-AZA treatment is lowest for cell lines with the highest base-level of methylation. However, although these observations hold on average and are significant statistically, there is still a remarkable degree of heterogeneity in the methylation patterns of the different genes in the different cell lines, with CIMP cells frequently showing hyperbolic trajectories, unlike the model predictions shown in Figure 4b. Another mark of heterogeneity in these data is evidenced by 10 of the 28 experiments not being modeled because they acted contrary to expectations, such as by following a decreasing pattern of methylation over time after 5-AZA treatment. The best example of this is a decrease in methylation of RASSF2-1 in the hct116 cell line, where it follows a smooth hyperbolic trajectory, but in the ‘wrong direction’. The curve for APC1 in the same cell line also goes in the wrong direction (although these data are very unstable). It almost suggests that a stochastic model might be useful that could allow a new target level for methylation after 5-AZA treatment, with the new target level for methylation not necessarily the same as the initial methylation level. A lack of close correspondence between initial and final methylation is seen in many of the other experiments.

***Authors’ response***: *We agree that a stochastic model is a useful tool given the degree of heterogeneity exhibited by the cell lines. Once more data becomes available, a stochastic description can be formulated and explored.*

The model used in this manuscript is called a negative feedback model (which leads to a final asymptote for methylation level) but a crucial part of the model (for non-CIMP cells) is the cascade of signaling molecules denoted by yi that through the kinetics of this cascade causes an initial delay in the increase of methylation. Although this model is plausible, there appears to be no data supporting these signaling components. Other models may be imagined that could produce a hyperbolic trajectory, such as a model with combined positive and negative feedback, e.g. see Pfeuty and Kaneko (2009). Still, the proposed model provides a reasonable hypothesis that can be tested by experiments that target the mechanisms which control DNA methylation levels in CIMP vs non-CIMP cells.

***Authors’ response***: *We agree with the referees that the proposed model is not the only possible explanation of the observed behavior. A separate study could look at the following general question: what combinations of feedback loops are consistent with the behavior exhibited by the cell lines. In the new text of the manuscript we included a discussion of these points, and added references.*

References: Otto SP, Walbot V., 1990. DNA methylation in eukaryotes: Kinetics of demethylation and de novo methylation during the life cycle. Genetics. 124 (2), 429–437.

Genereux DP, Miner BE, Bergstrom CT, Laird CD., 2005. A population-epigenetic model to infer site-specific methylation rates from double-stranded DNA methylation patterns. Proc Natl Acad Sci USA. 102 (16), 5802–5807.

Sontag, L.B., Lorincz, M.C. & Georg Luebeck, E., 2006. Dynamics, stability and inheritance of somatic DNA methylation imprints. Journal of Theoretical Biology, 242(4), pp.890–899.

Pfeuty B, Kaneko K. The combination of positive and negative feedback loops confers exquisite flexibility to biochemical switches. Phys Biol. 2009 Nov 12;6(4):046013

### Reviewer 2: Tomasz Lipniacki

The Authors investigate experimentally and theoretically dynamic CpG islands methylation in CIMP and non-CIMP colon cancer cells. CIMP (CpG island methylator phenotype) cells are characterized by higher level of CpG regions metylation than non-CIPM cells. The authors choose to study two CIMP lines (SW48 and RKO) and two non-CIMP lines (HT29 and HCT116) in which they focus on remethylation (following 5-AZA demethylation treatment) kinetics in 7 different sites. This gives 4 × 7 = 28 sites overall, which number is further reduced to 18 sites in which the remetylation kinetics follows assumed time profile.

The key observation is that (paradoxically) non-CIMP cells have faster rate of methylation recovery, although the remetylation process is delayed. In contrast in CIMP cells remethylation starts immediately but proceeds gradually at slower rate than for non-CIMP cells. The observation is in my opinion important.

Specific comments

1. My main objection is that the conclusions are based on very small data-set. For example from visual analysis of Figure 2 one can expect direction coefficients would be much different after removing single point corresponding to smallest base-level of methylation. Similarly in Figure 3 the non-CIMP and CIMP cells are grouped in a rather arbitrary way; again visually the htc116 could be easily assigned to CIMP group. I think the experimental part would be much stronger if the authors increase number of analyzed cells.

***Authors’ response***: *We agree with the referees that having more experimental data would make the conclusions stronger. We however claim that the trends reported here are statistically significant (we checked both Pearson rank correlation and the Spearman rank correlation, as reported in the text). Visual inspection in this particular case proves counterintuitive, because even after removing the point with smallest base-level of methylation, the results remains statistically significant. Further, the grouping of cell lines into CIMP and non-CIMP classes was not decided on the basis of the data presented here, but on the basis of previous research reported in the literature . The cell lines sw48 and rko are characterized as CIMP in the literature, while hct116 and ht29 are widely regarded as non-CIMP; a larger dataset would not alter this grouping. This is an important point, which has now been clarified in the text.*

2. It is very fair that Authors present and mention the lack of significant correlations between other characteristics of cells with respect to cell type (Figure 6, death rate, division rate etc.). However, this observation may suggest that “clusterization” shown in Figure 3 is not very meaningful.

***Authors’ response***: *Please see our previous remark with regards to (i) cell lines grouping and (ii) statistical significance of the correlation between the methylation rate and the base methylation level.*

3. In order to interpret experimental data Authors propose mathematical model. The main assumption is that remetylation in non-Cimp cells is subject to negative feedback (suppressing methylation) and time delay introduced by a sequence of events, finally releasing the feedback. In contrast, it is proposed that remetylation in CIMP cells starts without delay and proceeds at steady slow rate due to continuous activity of DNA methyltransferase. The above assumption allows to build a simple model “fitting” qualitatively the observed remethylation kinetics. In my opinion, however, the model structure is not supported (or contradicted) by presented data, and thus it is hard to make firm conclusions regarding the biological mechanisms of remethylation in CIMP and non-CIMP cells. For example one could also expect (purely by dynamical analysis) that the delay in remethylation is associated with the positive (not negative) feedback. Such systems frequently exhibit postponed activation.

***Authors’ response***: *We agree with the referees that the proposed model is not the only possible explanation of the observed behavior. A separate study could look at the following general question: what combinations of feedback loops are consistent with the behavior exhibited by the cell lines. The purpose of the present model is to demonstrate that feedback loops can be accountable for the observed counter-intuitive behavior. Further investigation of the mechanisms of methylation kinetics will inform the exact topology of the feedbacks. In the new text of the manuscript we included a discussion of these points, and added references to alternative models.*

***Reviewer’s response***: I am satisfied by the modifications and explanations provided by authors.

### Reviewer 3: Anna Marciniak-Czochra

This reviewer provided no comments for publication.

## Competing interests

The authors declare that they have no competing interests.

## Authors’ contributions

NK and DW designed the study, performed the statistical analysis, created and analyzed the mathematical model and wrote the paper. AG and RB performed the experiments. All authors read and approved the final manuscript.

## References

[B1] Vogelstein B Kinzler KW (Eds.): The genetic basis of human cancers 2002 New York: McGraw-Hill

[B2] Luebeck EG Moolgavkar SH Multistage carcinogenesis and the incidence of colorectal cancer Proc Natl Acad Sci USA 2002 99 15095 15100 10.1073/pnas.222118199 12415112 PMC137549

[B3] Moolgavkar SH Luebeck EG Multistage carcinogenesis and the incidence of human cancer Genes Chromosomes Cancer 2003 38 302 306 10.1002/gcc.10264 14566848

[B4] Knudson AG Jr Mutation and cancer: statistical study of retinoblastoma Proc Natl Acad Sci USA 1971 68 820 823 10.1073/pnas.68.4.820 5279523 PMC389051

[B5] Breivik J The evolutionary origin of genetic instability in cancer development Semin Cancer Biol 2005 15 51 60 10.1016/j.semcancer.2004.09.008 15613288

[B6] Cahill DP Lengauer C Yu J Riggins GJ Willson JK Markowitz SD Kinzler KW Vogelstein B Mutations of mitotic checkpoint genes in human cancers Nature 1998 392 300 303 10.1038/32688 9521327

[B7] Chow M Rubin H Clonal selection versus genetic instability as the driving force in neoplastic transformation Cancer Res 2000 60 6510 6518 11103821

[B8] Fodde R Kuipers J Rosenberg C Smits R Kielman M Gaspar C Van Es JH Breukel C Wiegant J Giles RH Clevers H Mutations in the APC tumour suppressor gene cause chromosomal instability Nat Cell Biol 2001 3 433 438 10.1038/35070129 11283620

[B9] Guo HH Loeb LA Tumbling down a different pathway to genetic instability J Clin Invest 2003 112 1793 1795 14679175 10.1172/JCI20502 PMC297004

[B10] Lengauer C Kinzler KW Vogelstein B Genetic instability in colorectal cancers Nature 1997 386 623 627 10.1038/386623a0 9121588

[B11] Loeb KR Loeb LA Significance of multiple mutations in cancer Carcinogenesis 2000 21 379 385 10.1093/carcin/21.3.379 10688858

[B12] Shih IM Zhou W Goodman SN Lengauer C Kinzler KW Vogelstein B Evidence that genetic instability occurs at an early stage of colorectal tumorigenesis Cancer Res 2001 61 818 822 11221861

[B13] Tomlinson I Different pathways of colorectal carcinogenesis and their clinical pictures Ann N Y Acad Sci 2000 910 10 18 discussion 18–20 10911902 10.1111/j.1749-6632.2000.tb06697.x

[B14] Tomlinson I Bodmer W Selection, the mutation rate and cancer: ensuring that the tail does not wag the dog Nat Med 1999 5 11 12 10.1038/4687 9883827

[B15] Tomlinson IP Novelli MR Bodmer WF The mutation rate and cancer Proc Natl Acad Sci USA 1996 93 14800 14803 10.1073/pnas.93.25.14800 8962135 PMC26216

[B16] Cahill DP Kinzler KW Vogelstein B Lengauer C Genetic instability and darwinian selection in tumours Trends Cell Biol 1999 9 M57 M60 10.1016/S0962-8924(99)01661-X 10611684

[B17] Boland CR Ricciardiello L How many mutations does it take to make a tumor? Proc Natl Acad Sci USA 1999 96 14675 14677 10.1073/pnas.96.26.14675 10611270 PMC33954

[B18] Brentnall TA Crispin DA Bronner MP Cherian SP Hueffed M Rabinovitch PS Rubin CE Haggitt RC Boland CR Microsatellite instability in nonneoplastic mucosa from patients with chronic ulcerative colitis Cancer Res 1996 56 1237 1240 8640805

[B19] Goel A Arnold CN Niedzwiecki D Chang DK Ricciardiello L Carethers JM Dowell JM Wasserman L Compton C Mayer RJ Characterization of sporadic colon cancer by patterns of genomic instability Cancer Res 2003 63 1608 1614 12670912

[B20] Iacobuzio-Donahue CA Epigenetic changes in cancer Annu Rev Pathol 2009 4 229 249 10.1146/annurev.pathol.3.121806.151442 18840073

[B21] Jones PA Baylin SB The fundamental role of epigenetic events in cancer Nat Rev Genet 2002 3 415 428 12042769 10.1038/nrg816

[B22] Laird PW Jaenisch R The role of DNA methylation in cancer genetic and epigenetics Annu Rev Genet 1996 30 441 464 10.1146/annurev.genet.30.1.441 8982461

[B23] Sharma S Kelly TK Jones PA Epigenetics in cancer Carcinogenesis 2010 31 27 36 10.1093/carcin/bgp220 19752007 PMC2802667

[B24] Sugimura T Ushijima T Genetic and epigenetic alterations in carcinogenesis Mutat Res 2000 462 235 246 10.1016/S1383-5742(00)00005-3 10767635

[B25] Issa JP CpG island methylator phenotype in cancer Nat Rev Cancer 2004 4 988 993 10.1038/nrc1507 15573120

[B26] Nosho K Irahara N Shima K Kure S Kirkner GJ Schernhammer ES Hazra A Hunter DJ Quackenbush J Spiegelman D Comprehensive biostatistical analysis of CpG island methylator phenotype in colorectal cancer using a large population-based sample PLoS One 2008 3 e3698 10.1371/journal.pone.0003698 19002263 PMC2579485

[B27] Toyota M Ahuja N Ohe-Toyota M Herman JG Baylin SB Issa JP CpG island methylator phenotype in colorectal cancer Proc Natl Acad Sci USA 1999 96 8681 8686 10.1073/pnas.96.15.8681 10411935 PMC17576

[B28] Toyota M Issa JP CpG island methylator phenotypes in aging and cancer Semin Cancer Biol 1999 9 349 357 10.1006/scbi.1999.0135 10547343

[B29] Ushijima T Watanabe N Shimizu K Miyamoto K Sugimura T Kaneda A Decreased fidelity in replicating CpG methylation patterns in cancer cells Cancer Res 2005 65 11 17 15665274

[B30] Watanabe N Okochi-Takada E Yagi Y Furuta JI Ushijima T Decreased fidelity in replicating DNA methylation patterns in cancer cells leads to dense methylation of a CpG island Curr Top Microbiol Immunol 2006 310 199 210 10.1007/3-540-31181-5_10 16909912

[B31] Boland CR Goel A Somatic evolution of cancer cells Semin Cancer Biol 2005 15 436 450 10.1016/j.semcancer.2005.06.001 16055343

[B32] Chen ZX Mann JR Hsieh CL Riggs AD Chedin F Physical and functional interactions between the human DNMT3L protein and members of the de novo methyltransferase family J Cell Biochem 2005 95 902 917 10.1002/jcb.20447 15861382

[B33] Denis H Ndlovu MN Fuks F Regulation of mammalian DNA methyltransferases: a route to new mechanisms EMBO Rep 2011 12 647 656 10.1038/embor.2011.110 21660058 PMC3128952

[B34] McAdams HH Arkin A Stochastic mechanisms in gene expression Proc Natl Acad Sci USA 1997 94 814 819 10.1073/pnas.94.3.814 9023339 PMC19596

[B35] Hermann A Gowher H Jeltsch A Biochemistry and biology of mammalian DNA methyltransferases Cell Mol Life Sci 2004 61 2571 2587 10.1007/s00018-004-4201-1 15526163 PMC11924487

[B36] Pradhan S Bacolla A Wells RD Roberts RJ Recombinant human DNA (cytosine-5) methyltransferase. I. Expression, purification, and comparison of de novo and maintenance methylation J Biol Chem 1999 274 33002 33010 10.1074/jbc.274.46.33002 10551868

[B37] Jair KW Bachman KE Suzuki H Ting AH Rhee I Yen RW Baylin SB Schuebel KE De novo CpG island methylation in human cancer cells Cancer Res 2006 66 682 692 10.1158/0008-5472.CAN-05-1980 16423997

[B38] Kanai Y Ushijima S Kondo Y Nakanishi Y Hirohashi S DNA methyltransferase expression and DNA methylation of CPG islands and peri-centromeric satellite regions in human colorectal and stomach cancers Int J Cancer 2001 91 205 212 10.1002/1097-0215(200002)9999:9999<::AID-IJC1040>3.0.CO;2-2 11146446

[B39] Lee PJ Washer LL Law DJ Boland CR Horon IL Feinberg AP Limited up-regulation of DNA methyltransferase in human colon cancer reflecting increased cell proliferation Proc Natl Acad Sci USA 1996 93 10366 10370 10.1073/pnas.93.19.10366 8816806 PMC38390

[B40] Saito Y Kanai Y Nakagawa T Sakamoto M Saito H Ishii H Hirohashi S Increased protein expression of DNA methyltransferase (DNMT) 1 is significantly correlated with the malignant potential and poor prognosis of human hepatocellular carcinomas Int J Cancer 2003 105 527 532 10.1002/ijc.11127 12712445

[B41] Turker MS The establishment and maintenance of DNA methylation patterns in mouse somatic cells Semin Cancer Biol 1999 9 329 337 10.1006/scbi.1999.0133 10547341

[B42] Slack A Cervoni N Pinard M Szyf M Feedback regulation of DNA methyltransferase gene expression by methylation Eur J Biochem 1999 264 191 199 10.1046/j.1432-1327.1999.00603.x 10447688

[B43] Pfeuty B Kaneko K The combination of positive and negative feedback loops confers exquisite flexibility to biochemical switches Phys Biol 2009 6 046013 10.1088/1478-3975/6/4/046013 19910671

[B44] Otto SP Walbot V DNA methylation in eukaryotes: kinetics of demethylation and de novo methylation during the life cycle Genetics 1990 124 429 437 2307364 10.1093/genetics/124.2.429 PMC1203935

[B45] Sontag LB Lorincz MC Georg Luebeck E: Dynamics, stability and inheritance of somatic DNA methylation imprints J Theor Biol 2006 242 890 899 10.1016/j.jtbi.2006.05.012 16806276

[B46] Genereux DP Miner BE Bergstrom CT Laird CD A population-epigenetic model to infer site-specific methylation rates from double-stranded DNA methylation patterns Proc Natl Acad Sci USA 2005 102 5802 5807 10.1073/pnas.0502036102 15827124 PMC556300

[B47] Mossman D Scott RJ Long term transcriptional reactivation of epigenetically silenced genes in colorectal cancer cells requires DNA hypomethylation and histone acetylation PLoS One 2011 6 e23127 10.1371/journal.pone.0023127 21829702 PMC3150411

[B48] Esteller M DNA methylation and cancer therapy: new developments and expectations Curr Opin Oncol 2005 17 55 60 10.1097/01.cco.0000147383.04709.10 15608514

[B49] Balaguer F Link A Lozano JJ Cuatrecasas M Nagasaka T Boland CR Goel A Epigenetic silencing of miR-137 is an early event in colorectal carcinogenesis Cancer Res 2010 70 6609 6618 10.1158/0008-5472.CAN-10-0622 20682795 PMC2922409

[B50] Yang AS Doshi KD Choi SW Mason JB Mannari RK Gharybian V Luna R Rashid A Shen L Estecio MR DNA methylation changes after 5-aza-2’-deoxycytidine therapy in patients with leukemia Cancer Res 2006 66 5495 5503 10.1158/0008-5472.CAN-05-2385 16707479

[B51] Yang AS Estecio MR Doshi K Kondo Y Tajara EH Issa JP A simple method for estimating global DNA methylation using bisulfite PCR of repetitive DNA elements Nucleic Acids Res 2004 32 e38 10.1093/nar/gnh032 14973332 PMC373427

